# Narrative review of the epidemiology, diagnosis and pathophysiology of pelvic organ prolapse

**DOI:** 10.1590/S1677-5538.IBJU.2018.0581

**Published:** 2020-01-13

**Authors:** Adi Y. Weintraub, Hannah Glinter, Naama Marcus-Braun

**Affiliations:** 1 Department of Obstetrics and Gynecology, Soroka University Medical Center, Faculty of Medicine, Ben-Gurion University of the Negev, Beer-Sheba, Israel; 2 Department of Obstetrics and Gynecology, Ziv Medical Center, Faculty of Medicine, Bar-Ilan university, Safed, Israel

**Keywords:** Epidemiology, physiopathology [Subheading], Pelvic Organ Prolapse

## Abstract

The exact prevalence of pelvic organ prolapse is difficult to establish. The anatomical changes do not always consist with the severity or the symptoms associated with prolapse. There are many risk factors associated with pelvic organ prolapse and this review aims to identify the epidemiology and pathophysiology while looking at the known risk factors for pelvic organ prolapse. PubMed search involved a number of terms including: epidemiology, risk factors, reoccurrence indicators, management and evaluation. Several risk factors have been associated with pelvic organ prolapse, all contribute to weakening of the pelvic floor connective tissue/collagen, allowing the pelvic organs to prolapse through the vaginal walls. Among the risk factors are genetic background, childbirth and mode of delivery, previous hysterectomy, menopausal state and the ratio between Estrogen receptors. The “Integral theory” of Petros and the “Levels of Support” model of Delancey enable us to locate the defect, diagnose and treat pelvic organ prolapse.

The currently available demographic data is not reliable enough to properly estimate the true extent of pelvic organ prolapse in the population. However, standardization of the diagnosis and treatment may significantly improve our ability to estimate the true incidence and prevalence of this condition in the coming years.

## INTRODUCTION

Pelvic organ prolapse (POP) is a disturbing problem, which affect many women and their quality of life (1). In the literature, there is a discrepancy regarding the true prevalence of POP which can be related to the type of study performed (2–4). While studies presenting anatomical prolapse observed during gynecological examination describe the prevalence of POP up to 50%, other studies which involve only questionnaires of bothersome symptoms, describe much lower prevalence (2, 3). The actual number of women that undergo intervention for POP seems to be similar to the prevalence described in telephonic surveys (5). Bulge symptoms and other associated prolapse symptoms are more significant than the anatomical changes that can be seen during gynecological examination.

Although many factors were described in association with POP, the relationship between the risk factors themselves is not clear and not always well understood. Weakness of the endopelvic fascia is the main factor in the etiology of POP and all the known risk factors actually cause weakness and damage of the fascia and therefore may result in herniation of the organs and prolapse (6).

**Figure 1:**
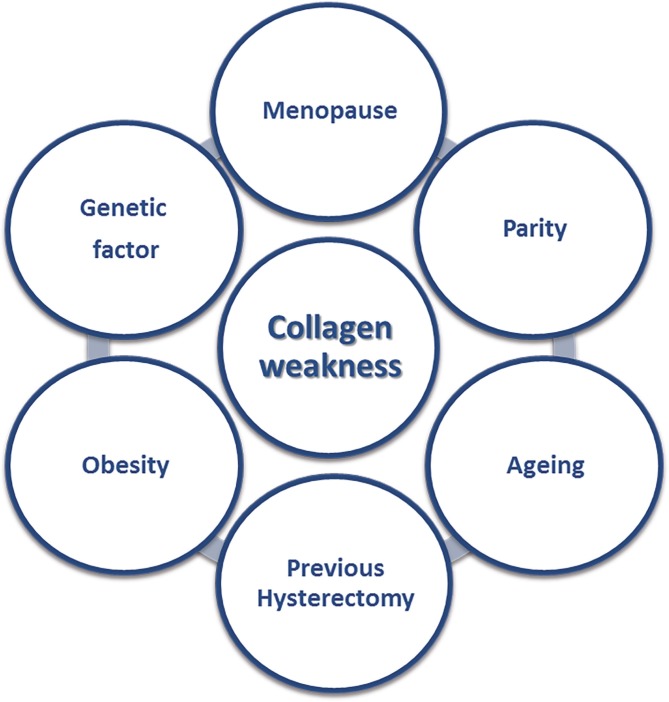
Risk factors for pelvic organ prolapse, causing collagen weakness.

The aim of this narrative review is to describe the actual prevalence of symptomatic POP based on the literature and to try to relate the known risk factors (Figure-1) to the pathophysiology of POP. Understanding the pathophysiology and risk factors, may lead to better diagnosis and treatment.

### Methodology

The content of this article was compiled through a literature review of peer reviewed journal articles and studies related to the topic of pelvic organ prolapse (POP). PubMed was the primary database used to search for journal articles and studies for the review. In order to prepare this review we performed a Medline search for English articles using the following key words: “pelvic organ prolapse”, “cystocele”, “rectocele”, “apical prolapse”, “epidemiology”, “risk factors”. We reviewed the article's references as well. We strained to include the most recent articles from the best existing journals for this update of the literature on this topic. A total of 55 references were used to review the epidemiology, pathophysiology, and management of pelvic organ prolapse.

### Epidemiology and demographic characteristics of pelvic organ prolapse

Pelvic organ prolapse is defined as a protrusion or herniation of the pelvic organs through the vaginal walls and pelvic floor. It is a common condition that affects many women. However, the exact prevalence is difficult to establish. It is frequently quoted that about 50% of all women will develop POP, but this refers only to the anatomical changes and does not reflect the severity of pro-lapse or the symptoms associated with prolapse. Therefore, the prevalence of symptomatic POP is actually much lower (1).

The reported prevalence of POP is highly varied according to different studies and is found to be anywhere between 3% and 50% (2–4). These wide variations are due to differences in study design, inclusion criteria, and accompanying indicator symptoms used among studies. For example, studies that are based on telephone surveys without a gynecological examination rely on the subjective bulge sensation reported by women and estimate the prevalence of POP to be between 2.9% and 8.3% (2, 3). In contrast, in other studies that are based on an objective gynecological examination with no regard to women's subjective symptoms, the prevalence of any POP is reported to as high as 50%. There is more than one evaluation method used in order to quantify the extent of any individual prolapse. Grading from 0-4 describes the descent of the prolapse from minimal prolapse to the greatest possible descent. In these studies, most of the women reported POP grade 1 or 2 with the rate of POP grade 3 being only 2%-3% (1, 4). Although, telephonic surveys cannot replace gynecological examination, it seems that they better describe symptomatic POP and are therefore important.

Pelvic organ prolapse can be defined by the descent of the compartment according to the vaginal segment and is divided into anterior, posterior, and apical vaginal compartments. Data regarding the type of prolapse or the compartment most often affected are available from epidemiological studies as well as from studies reporting preoperative evaluation. It has been found that prolapse of the anterior compartment occurs most frequently among the three types and is reported to be twice as prevalent as prolapse of the posterior compartment and three times more prevalent than prolapse of the apical compartment (7, 8). It should be noted that POP is a dynamic condition and that to a certain extent, two thirds of women have a combined prolapse of all three compartments. The prevalence of prolapse of the vaginal cuff following hysterectomy was reported to be as high as 6%-12% (9).

Among women having symptomatic POP, the age distribution increases dramatically. Women between the age of 20-29 account for 6% of the women suffering from POP, while women aged 50-59 years account for 31% with POP and close to 50% of women with POP are aged 80 years or older (10). With increased longevity and an increase in the demographic of women over 65 years, it is expected that in the near future POP will become a major health concern. Wu et al. have estimated that in the USA in 2050, the prevalence of women suffering from symptomatic POP will increase to 46%, which translates to over 5 million individuals (11).

The age association of POP is further revealed by studies identifying those who seek medical consultation and care for their symptoms. The average age of women seeking medical consultation for symptomatic POP is 61 (10). According to the demographic study performed by Luber et al. (12), there is a positive association of increasing age of women and those who seek medical help for POP. The rate of women aged 30-39 who seek medical help for POP is 1.7/1000. The rate increases among women aged 60-69 to 13.2/1000. The highest rate among those seeking medical consult for symptomatic POP was reported in women aged 70-79 and is as high as 18.6/1000 (12).

Other studies that give insight regarding the prevalence of POP are those reporting data on patients who have undergone POP reconstruction surgeries. From these studies it appears that a woman's lifetime risk of undergoing a surgery for POP or stress urinary incontinence (SUI) is 11%-20% (10, 13, 14). However, these data sets do not indicate true prevalence rates of POP for a number of reasons. Many women that suffer from POP may be asymptomatic, or not seek medical attention for other reasons. In addition, many women with POP that seek medical attention are managed conservatively and are not treated with surgery. Lastly, there is inconsistency between studies regarding the grade of POP that requires surgical intervention. Therefore, there is a lack of standardization between the different reports.

As with those who seek medical care and consultation, the prevalence and incidence of POP reconstructive surgery also increases with age (10). By the age of 80 years, the lifetime risk of a woman in the USA undergoing at least one surgery for POP is 6.3% and the risk of recurrent surgery is 30% (13). In Australia, a woman's risk of undergoing at least one surgery for POP is threefold higher at 19% (14). This difference may be explained in part by differences in surgical practice, incorporation of new surgical techniques, medical insurance coverage, and different cultural perceptions of quality of life (QoL). The annual rate of POP surgery in the USA is 1.5-1.8/1000 women with the highest rates reported among women aged 60-69 years. This is comparable to the rate of women referring to medical help due to POP (5).

Another important epidemiological indicator is the rate of recurrent POP and the need for recurrent surgery. This data is unreliable and the prevalence is not completely clear because not every recurrence is symptomatic. In addition, the evaluation of POP that determines the need for repeat surgery has changed in recent years. While in the past prolapse recurrence was considered a surgical failure, in recent years, symptom relief and improved QoL are recognized as the determining factors for surgical success. There is approximately a 30% recurrent prolapse rate following POP repair surgery (13). However, this approximation does not take into account the stage of prolapse or presence of symptoms. Recently, the two main international organizations in urogynecology, the International Continence Society (ICS) and the International Urogynecological Association (IUGA) have presented a joint report on the terminology for reporting outcomes of surgical procedures for POP that incorporates anatomical outcomes as well as subjective patient's symptoms, QoL and satisfaction (15).

### Risk factors and Pathophysiology of POP

Several risk factors have been associated with POP. All risk factors contribute to weakening of the pelvic floor connective tissue/collagen, causing the pelvic organs to prolapse through the vaginal walls and pelvic floor (Figure-1). There are predisposing, non-modifiable factors including race, gender and genetic make-up. Other promoting risk factors for which intervention or prevention can be of benefit, include occupation, obesity, smoking, and infection, and there are inciting risk factors such as childbirth causing muscle, connective tissue, vascular and neural damage (16).

### a) BMI/Obesity

Obesity directly affects symptoms of pelvic organ prolapse. A chronic increase in intra-abdominal pressure, nerve damage and co-morbidities of obese individuals all contribute to pelvic floor dysfunction (17, 18). Intra-abdominal pressure causes excessive strain on pelvic structures, including the pudendal nerve. Co-morbidities such as diabetes contribute to poor tissue features through neuropathy and genetic background and joint hypermobility.

### b) Genetic

It is well established that there is a genetic predisposition for POP, independent of all other risk factors that may impact or aggravate the condition. In women with a family history of prolapse there is a 2.5-fold increased incidence of POP compared with the general population (19). Many women with POP report having relatives with POP, urinary incontinence and/or an abdominal or inguinal hernia (20). In addition, younger women with POP have a higher incidence of POP among first-degree relatives than those who develop POP at an older age (21).

The association between POP and other conditions with impaired collagen quality has been shown in many studies, which further implies a genetic predisposition. The incidence of collagen diseases such as varicose veins and joint hypermobility was increased in women with POP and in a recent meta-analysis of 39 studies, joint hypermobility as an indicator for POP was determined to be clinically relevant (22).

The strength of collagen, the main component of the body's connective tissue, and specifically of the pelvic floor fascia and ligaments, is determined by genetic factors. The type of collagen and the body's ability to replace damaged collagen with collagen that is strong and of high quality is also determined by genetic factors (23).

Several studies have attempted to identify and characterize the genes that are responsible for POP. In a recent meta-analysis it was found that collagen type 3 alpha 1 (COL3A1) rs1800255 genotype AA was significantly associated with POP in an Asian and Dutch population compared with a reference genotype population (OR 4.79; 95% CI 1.91-11.98; P <0.001) (24). Other studies investigated different populations; however, they were limited by a small sample size, preventing them from drawing meaningful conclusions. With the advances seen in molecular biology and the possibility to decipher entire genes it is conceivable that in the near future scientists will find the genes responsible for collagen strength and therefore those that predispose POP.

### a) Obstetrical and gynecological history

Parity: Multi-parity may be the strongest predisposing factor to POP. Women with one child show a fourfold increased likelihood to experience POP requiring hospital attention and those with two children an 8.4 times greater likelihood, compared with nulliparous women (25). Interestingly, while parity is an established risk factor for primary POP, it is not a risk factor for recurrence (26).

Mode of delivery and obstetrical trauma: Vaginal delivery has an extensive role in pelvic floor damage and the eventual development of POP. It is understood that most of the damage to the pelvic floor occurs during first and second deliveries (27). Pelvic floor imaging studies have demonstrated the “Ballooning” phenomenon after delivery. This phenomenon describes the widening of the pelvis during the Valsalva maneuver that represents the expansion of the levator-ani muscles. This phenomenon can be demonstrated after delivery using a 3D ultrasound and in a vaginal examination (28).

Although rare, POP in women with no vaginal deliveries is possible. Cesarean section serves as a protective factor from POP if there was no additional vaginal delivery (29). Instrumental deliveries increase the risk for POP, forceps delivery in particular (30).

As an added obstetrical risk factor, cervical elongation is also reported to affect approximately 40% of women with uterine prolapse. The cervical length in women with uterine prolapse was measured to be about 36% longer than in women without uterine prolapse (31).

Hysterectomy: An increased risk for central compartment prolapse is noted in women who have undergone hysterectomy as compared with women with in situ uterus. Possible explanations for this observation include: intraoperative damage to the pelvic connective tissue, injury to the pelvic blood supply and innervation, as well as not enough emphasis placed on the secure fixation or suspension of the vaginal apex in many hysterectomy procedures. According to a cohort study that evaluated 160.000 women following hysterectomy, the risk of developing POP was 3.2% compared with only 2% in controls (32). Compared with non-hysterectomized controls, the overall Hazard ratios (HR) for prolapse surgery was 1.7 (95% CI, 1.6 to 1.7) with the highest risks observed in women having had a vaginal hysterectomy (HR 3.8; 95% CI, 3.1 to 4.8). However, it should be noted that the indication and type of hysterectomy were not reported. It is therefore unclear what is the exact proportion of women who have undergone a vaginal hysterectomy due to previous POP. According to other studies it is clarified that the risk of developing an apical prolapse following a vaginal hysterectomy due to POP is five-fold higher even if a prolapse correction was performed in the primary surgery (33).

### a) Menopause

While advanced age is a risk factor for POP as discussed in earlier sections, and menopause is a consequence of age, there is a straight association between menopause and an increased risk for POP that is independent of age or parity (34, 35). The hormonal changes in menopause cause a drop in the systemic estrogen concentrations, and a hypoestrogenic environment in the pelvic organs contributes to alterations in the composition and strength of collagen (36).

Studies that evaluated the influence of estrogen and of selective estrogen receptor modulators (SERM) on the development of POP have shown conflicting results. According to some studies, Raloxifene and Tamoxifen have worsened the severity of POP as compared with estrogen and placebo (37, 38). In contrast, a prospective study that investigated the impact of Raloxifene treatment on the development of POP showed a 50% decrease in surgical intervention for POP in a group of post-menopausal women (39). An increase in the rate of POP was demonstrated with the use of other drugs of the SERM family such as Levormeloxifene and Idoxifene and POP has even been stated as a side effect of these medications (40, 41).

The impact of estrogen on the tissue is not only dependent on the estrogen concentrations but also on the expression of estrogen receptors. Estrogen and estrogen receptors modify genes that encode growth factors in the extracellular matrix. During menopause, changes in the concentration and quality of collagen, connective tissue morphology and the role of estrogen in the metabolism of collagen are all indicators of the involvement of estrogen in the development of POP (36). The concentration of collagen in the vagina is determined by the equilibrium between collagen metabolism and catabolism. Estrogen receptors can be found among other tissues, in the nucleus of connective tissue cells, smooth muscle cells in the bladder trigone, in the vaginal mucosa, in the levator-ani muscle and in the utero-sacral ligaments, of which the utero-sacral and the cardinal ligaments are essential components of organ support (42). In post-menopausal women with POP, significantly lower concentrations of serum estrogen and lower concentrations of estrogen receptors in the pelvic floor ligaments were found as compared to women without POP (43, 44).

The type of estrogen receptors is also a factor associated with the development of POP. In women with POP an alteration in the ratio of alpha and beta estrogen receptors was noted. In post-menopausal women with POP a 1.5-2.5 fold decrease in alpha estrogen receptors was found. Moreover, in pre-menopausal women without POP, an increase in beta estrogen receptors was measured as compared to women with POP (45).

The apparent influence of estrogen and SERM on the synthesis of estrogen receptors may explain the contradicting association between SERM and the incidence of POP, most likely by altering the ratio between alpha and beta estrogen receptors. Much more research is needed in order to fully understand these associations.

In conclusion, conditions that cause an increase in intra-abdominal pressure such as chronic cough and constipation, obesity, modifiable risk factors such as a lifestyle or occupations that require lifting heavy loads and medical conditions that involve the connective tissue such as Ehlers-Danlos syndrome or Marfan syndrome are considered risk factors for POP (46–48).

While individual risk factors affecting the prevalence of POP such as age, vaginal deliveries and race are well identified, comorbidities such as DM together with hypertension must be considered in the development of the condition (49).

### Identification and Management of POP

The current understanding of the pelvic floor is based on the work of two modern anatomists: Peter Petros and John Delancey. These two researchers have studied the pelvic floor extensively and have incorporated in their studies advanced dynamic imaging techniques.

### The “Integral Theory”

The “Integral Theory” represents the foundation of our current knowledge of the development of POP. Published by Peter Petros in 1990, it is the cornerstone of our understanding of the pathogenesis of prolapse as well as the definition of the treatment (6). According to this theory, POP and its related symptoms result from over-laxity of the vaginal connective tissue or its supporting ligaments. The integral theory includes four components: normal function, dysfunction, diagnosis, and treatment (50).

**Figure 2*:**
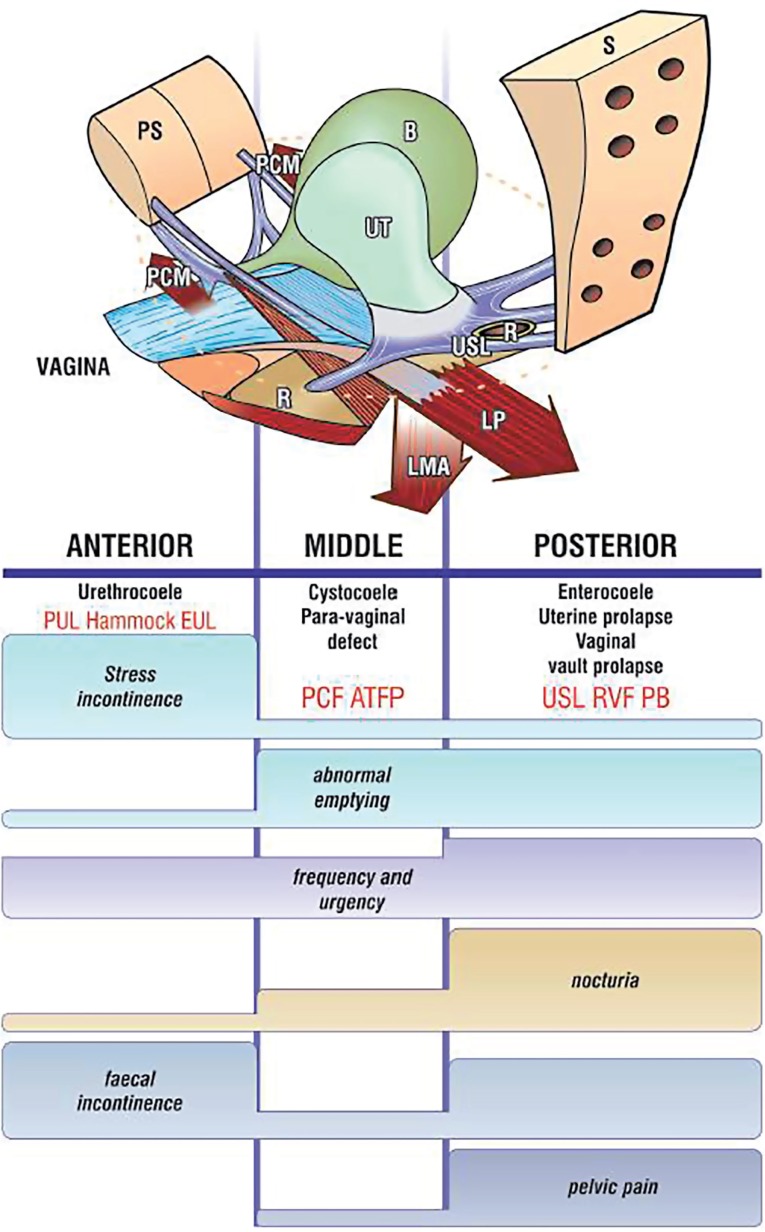
Schematic diagram of the pelvis organs, ligaments, muscles and the connection between them according to the integral theory of Peter Petros. *source www.intergraltheory.com, Petros with permission **PUL** = Pubourethral ligament; **PCF** = Pubocervocal fascia; **ATFP** = Arcus tendineus fascia pelvis; **CL =** Cardinal ligament/cervical ring; **USL** = Uterosacral ligament; **RVF** = Rectovaginal fascia; **PB** = Perineal body

The bladder, vagina, and rectum are pelvic organs held in place by supporting ligaments including the pubo-urethral (PUL) and pubo vesical (PVL) ligaments, the utero-sacral ligaments (USL), the cardinal ligaments, and the arcus tendineus fascia pelvis (ATFP) (Figure-2). The pelvic floor fascia joins these ligaments and the perineal body. The main component of the pelvic floor fascia and of the ligaments is collagen. The pelvic floor muscles pull back the pelvic organs and the pelvic floor fascia in three different directions providing them with support and maintaining their form and strength (Figure-1). The pelvic floor ligaments and fascia can be depicted as a suspension bridge, where the strength of the bridge is dependent on the strength of the ligaments. Injury or damage to one of the ligaments will bring about the collapse of the bridge. Likewise, injury or weakening of one of the pelvic floor ligaments will cause a herniation of the pelvic organs according to the location of the affected ligament. According to the integral theory the pelvis is divided into three areas: anterior, middle, and posterior, in which connective tissue laxity affects the organs and their function (Figure-1). It should be noted that while there may be severe symptoms of prolapse, this may not correlate with the actual severity of prolapse and may occur with minimal prolapse (51, 52).

According to the integral theory a discreet examination is performed by area of the pelvic floor in order to evaluate the damage and to focus the treatment to a specific area (50). Moreover, it helps us to understand the symptoms and the location of the prolapse as well as to understand that the main injury to the pelvic floor is in the pelvic floor connective tissue, namely, the pelvic floor fascia and the ligaments.

### The “Levels of Support” model

John Delancey has described the levels of support model, according to which support to the pelvic organs is divided into three levels (53, 54). Delancey, like Petros, acknowledges that the connective tissues, the pelvic floor fascia and the pelvic floor ligaments, are responsible for holding the pelvic organs in place and that injury to each level of support causes damage to a specific area. Additionally, Delancey's model enables the diagnosis and treatment of the prolapse by level of injury. Delancey has used advanced imaging technologies to predict prolapse. Specifically, using magnetic resonance imaging (MRI), he studied the dynamics of the supporting ligaments at rest and during the Valsalva maneuver, and the respective change in prolapse (55).

Petros and Delancey's work enable us to locate, diagnose and treat POP but do not provide answers regarding the causes for weakening of the connective tissue and pelvic floor ligaments. It is still not entirely understood why some multiparous women do not report prolapse symptoms and may have no or only minimal POP, while others will suffer symptomatic prolapse at a young age after only one delivery. The answers regarding the causes of pelvic floor connective tissue weakening and development of vaginal herniation may be derived from a closer look at prolapse risk factors.

### Summary

From a public health point of view, POP has a tremendous economic burden on health systems. The increase in life expectancy and the movement towards improved QoL, contribute not only to the increase in the prevalence of POP but also to the increase in prevalence of women seeking treatment and solutions for their symptoms. The currently available demographic data is not reliable enough to properly estimate the true extent of POP in the population. However, a continuing joint effort of the international associations (IUGA and ICS) in standardization of the diagnosis and treatment of POP, may significantly improve our ability to estimate the true incidence and prevalence of this condition in the coming years.

The understanding that the pelvic floor relies on the pelvic floor fascia and ligaments for its support enables us to identify the specific injury causing the prolapse and to treat it accordingly. We owe this understanding to the works of Peter Petros and John Delancy. However, the causes for weakening of the connective tissue and pelvic floor ligaments is still unclear and answers may be found while looking closer at the risk factors. It is evident that there is a strong genetic basis for POP. Identifying the genes responsible for the quality of collagen will enable us to council high risk nulliparous women regarding possible preventive measures including physiotherapy, avoidance of strenuous activity and even elective cesarean delivery. A hypoestrogenic environment especially in post-menopausal women has a significant role in the development of POP. This is illustrated by medications from the SERM family impacting the development or prevention of POP. Continuing research and bettering our understanding of the role of estrogen receptors and the change in ratio between the types of these receptors may lead to the development of new drugs to reinforce damaged collagen, prevent, or even reverse POP.
